# Medicine Insufficient Evidence for the Efficacy of Massage as Intervention for Autism Spectrum Disorder: A Systematic Review

**DOI:** 10.1155/2022/5328320

**Published:** 2022-09-24

**Authors:** Hui Ruan, Wichai Eungpinichpong, Hua Wu, Minggui Shen, Aijiao Zhang

**Affiliations:** ^1^Faculty of Graduate School, Khon Kaen University, Khon Kaen, Thailand; ^2^Faculty of Physical Education, Hainan Normal University, Haikou, China; ^3^PT Division of Physical Therapy, Faculty of Associated Medical Sciences, BNOJHP Research Center, Khon Kaen University, Khon Kaen, Thailand; ^4^Hainan Special Education School, Haikou, China

## Abstract

The efficacy of massage therapy in the treatment of children with autism spectrum disorder (ASD) remains unclear. This study systematically reviewed the impact of massage therapy on children with ASD according to the preferred reporting items for systematic reviews and meta-analyses (PRISMA) declaration guidelines. A literature search of the PubMed, Web of Science, Science Direct, Scopus, Google Scholar, and China National Knowledge Infrastructure (CNKI) electronic databases from inception to December 20, 2020, was conducted using the term “autistic/autism” along with one of the following terms, “massages,” and “Tui na.” The risk of bias was assessed using the Cochrane risk of bias Tool. Eight randomized controlled trials examining the impact of massage on children with ASD were included. Interventions combining Qigong massage or Tui na with the control group treatments from once a day to twice a week, for a duration of 15–30 mins, and lasting for six weeks to five months were the main interventions. All reviewed studies reported significant improvement in children with ASD who received massage, especially in the sensory domain, and that massage in combination with control treatment was superior to control treatment alone. However, the overall quality of the available studies is poor with a high degree of heterogeneity. The majority of studies showed a high risk of bias with poor study design, inconsistency in massage protocols, and subjective outcome measures. Assessment bias was a common weakness of these studies. Therefore, there is insufficient evidence to conclude that massage is effective for ASD. Future studies should include large sample sizes, incorporate double-blind designs, employ appropriate outcome measures, and allow for long observation and follow-up periods. Furthermore, consensus must be reached on standardized treatments and additional therapies in order to provide better quality evidence for the treatment of ASD.

## 1. Introduction

According to global statistics, the incidence of autism spectrum disorder (ASD) is increasing with an estimated prevalence of ∼1.5% in developed countries [1]. An updated Centers for Disease Control and Prevention report indicates that one in forty four eight-year-old children is estimated to have ASD in the United States [2]. ASD is a neurodevelopmental disorder that is distinguished by core deficits in social interaction and communication, as well as restricted and repetitive patterns of behavior [3]. Sensory processing is significantly associated with social and behavioral functioning [4, 5] and is listed as a criterion for diagnosis of ASD in the Diagnostic and Statistical Manual of Mental Disorders [3]. Studies have reported that the frequency of sensory processing difficulties in ASD populations ranges from 43% to 95%, which is significantly higher than that of in the neurotypical groups [6, 7], with a higher prevalence of sensory underresponsivity, overresponsivity, and sensation seeking in ASD populations [8]. A recent study of 25,627 people with ASD found that 74% of children aged 4 to 8 years old showed atypical responses to sensory stimuli with impaired tactile processing frequently associated with social dysfunction [9].

There are currently no approved medications to treat the core symptoms of ASD due to its complex etiology and heterogeneity [10]. The refractory and life-long nature of the disorder places a heavy burden on families and society [11]. Early screening and intervention are important for promoting positive development and improving outcomes for children with ASD [12, 13]. This has prompted a demand for complementary and alternative medicine (CAM) treatments for children with ASD [14]. Massage includes many different methods or techniques but usually involves another person applying appropriate pressure to various parts of the body. Traditional Chinese massage (or Tui na), traditional Indian massage, and Swedish massage are the most common types of massage [15, 16]. Massage therapists use their hands, forearms, and elbows for pushing, kneading, pressing, patting, and other operating techniques. Massage is commonly practiced in some countries for the treatment of musculoskeletal and neurological disorders and it has been shown to have a positive effect on relieving pain, eliminating fatigue, improving mood, and improving some clinical disorders [17]. It has been suggested that massage may provide beneficial effects for children with ASD [18, 19]. Data from the Boston Developmental Medicine Center at Children's hospital showed that 11% of children with ASD were treated with a massage from 1997 to 2003 [20]. Consequently, massage therapy has high uptake and acceptability according to parents' perception. However, massage therapy is still not incorporated into evidence-based Practices for ASD treatment [21] as a result of insufficient evidence. Thus, the efficiency of massage intervention for ASD remains understudied. To our knowledge, there have been two reviews that focused on massage therapy for ASD. Lee et al. [22] reviewed six studies in 2011 and concluded that there was limited evidence of the effect of massage treatment on ASD. These studies, however, had small sample sizes and a high risk of bias. Walaszek [19] summarized the application and efficacy of selected massage treatments on ASD in a narrative review, but the cited reviews contained sparse research on traditional Chinese massage and could not provide sufficient evidence to support the effects of massage therapy on ASD.

Herein, we seek to evaluate massage therapy as a viable treatment option for ASD and to provide recommendations for future massage interventions. Our study summarizes the results of past and current research on the effectiveness of massage intervention in controlled trials in children with ASD. Five research questions guided the study are

The primary research questions are as follows:
  1. To date, what are the main kinds of massage treatments for children with ASD?
  2. What are the effect of these massage interventions in improving symptoms in ASD children?
  3. What are the characteristics of the tools used to evaluate the effects of massage intervention on children with ASD?
The secondary research questions are as follows:
  1. What are the possible mechanisms of massage that may affect ASD children?
  2. What is the acceptability and limitation of massage intervention?

## 2. Materials and Methods

### 2.1. Study Design

This review followed preferred reporting items for systematic reviews and meta-analyses (PRISMA) guidelines [23]. The PICOS framework (participants, interventions, comparisons, outcomes, and study design) [24] was used to identify key study concepts and conduct the search process in the research. The review protocol was prospectively registered on the PROSPERO database (protocol ID: CRD42020212853). Ethical approval or informed consent was not required.

### 2.2. Search Strategy

We searched six electronic databases including PubMed, Web of Science, Science Direct, Scopus, Google Scholar, and China National Knowledge Infrastructure (CNKI). The search included peer-reviewed manuscripts published between January 1990 and December 2020 in English and Chinese. Our search strategy focused on population (children with ASD), intervention (any type of massage intervention or massage combined with other nondrug therapies used in a control group for more than 6 weeks), comparator (control group with usual daily life or receiving a nondrug treatment), outcome (e.g., a core symptom of ASD), and study design (randomized controlled trial). The following keywords were searched in combination with “OR” and “and”: (1) PubMed (“autism” [Title/Abstract] or “autistic” [Title/Abstract]) and (“massage” [Title/Abstract] or “Tui na” [Title/Abstract]); (2) Web of Science (((AB = (autism)) or AB = (autistic))) and AB = (message) or (Tui na); (3) Science Direct Title, abstract, keywords: (“autism” or “autistic”) and (“massage” or “Tui na”); (4) Scopus TITLE-ABS-KEY (“autism” or “autistic”) and (“message” or “Tui na”); (5) Google Scholar: allintitle: autism massage, allintitle: autistic massage; and (6) CNKI: (Title-Abstract-Key = Zi Bi Zheng) or (Title-Abstract-Key = Gu Du Zheng))) and (Title-Abstract-Key = An Mo) or (Title-Abstract-Key = Tui Na).

### 2.3. Eligibility Criteria

Articles were included if they (a) reported the findings from a randomized controlled trial (RCT), (b) cited massage as a main or as part of a mixed intervention, (c) quantified outcome measures in children with ASD symptoms, (d) involved children with ASD between 2 and 18 years old at baseline, and (e) were published in either English or Chinese. The following were the exclusion criteria: (a) quantitative cross-sectional studies, (b) qualitative studies, (c) studies that recruited fewer than 10 people, (d) nonhuman studies, (e) other clinical populations, and (f) thesis, conference paper, and articles without full text.

### 2.4. Study Selection and Data Extraction Strategy

Two of our groups (H.R. and H.W.) independently screened titles, abstracts, and full-text articles for potential inclusion. Each important study feature (e.g., first author, publication year, sample size, age, study design, characteristic/s of massage intervention, and outcome) was extracted separately. Any quantified outcomes about autism symptom changes before and after intervention were collected. Intervention characteristics and side effect reports were also collected. For incomplete data, the author was contacted by e-mail for further information. In the case of disputes, a third researcher (W.E) made the final decision. All retrieved studies were inserted into Zotero 5.0 software (Windows, CC-BY-SA 4.0) to manage the data.

### 2.5. Assessment of Risk of Bias

Each included study's risk of bias was assessed using the Cochrane risk of bias tool [25] recommended by the Cochrane handbook [26]. Two authors (H.R. and H.W.) evaluated individual studies independently in five domains: the process of randomization, deviation from the intended interventions, missing outcome data, outcome measurement, and selection of the reported result. The risk of bias in each domain was divided into three levels, “low risk of bias,” “some concerns,” and “high risk of bias.” If the evaluation result in all fields was “low risk,” then the overall risk of bias was classified as “low risk.” If the evaluation results in some fields were “some concerns” and there were no “high risk” fields, the overall bias risk was “some concerns.” The overall bias risk was classified as “high risk” when at least one field was assessed as “high risk.” The third researcher (W.E) evaluated disputed results.

## 3. Results

### 3.1. Search Results

**Figure 1:**
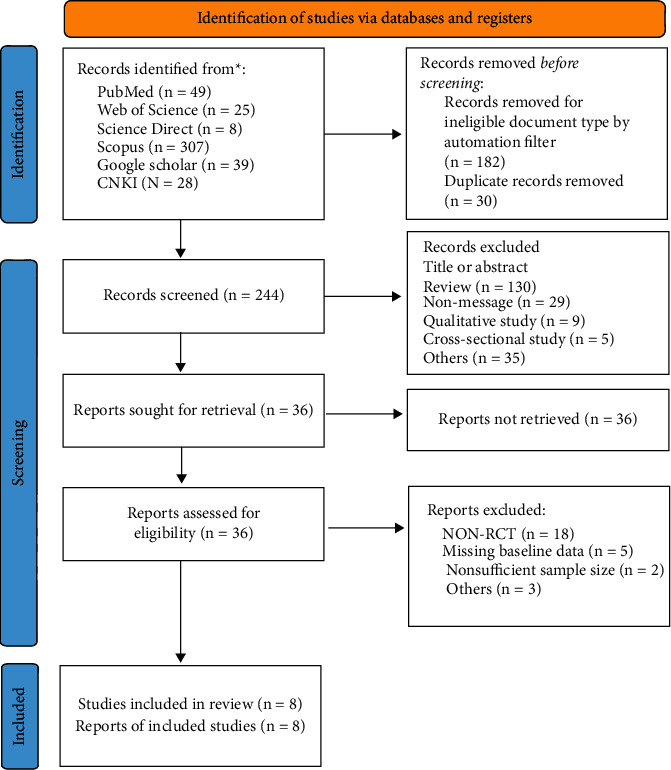
PRISMA flow diagram of the study selection process.

The database search identified 456 studies: 49 from PubMed, 25 from Web of Science, 8 from Science Direct, 307 from Scopus, 39 from Google Scholar, and 28 from CNKI (PRISMA flow diagram of the study selection process in Figure 1). Only eight studies that involved massage as an intervention in children with ASD met our requirements and were selected.

### 3.2. Study and Participant Characteristics

The sample sizes of the studies ranged from 15 to 84 children with ASD, aged 2–8 years old. Qigong massage [27–30] and Tui na [31–34] were the main interventions. Control groups included treatments such as special education programs [27–30] and acupuncture [31, 32], and experimental groups were set up with massage interventions in addition to the treatment provided in the corresponding control group. The duration of the interventions varied from 6 weeks [33] to 5 months [27–29], the frequency of treatment ranged from daily to twice per week, and section times ranged from 15 mins to 30 mins per section [27–30]. The outcome measures included the Autism Behavior Checklist (ABC), Childhood Autism Rating Scale (CARS), Sensory Profile, Pervasive Developmental Disorders Behavior Inventory (PDDBI), Autism Parenting Stress Index (APSI), and the Sense and Self-Regulation Checklist (SSC). Further information for all individual RCTs is summarized in Table 1. Two studies [29, 30] obtained Institutional Review Board approval before conducting the study, and one [30] was a registered clinical trial. Two studies had follow-ups five months after treatment [22, 24].

### 3.3. Quality Assessment of the Included Studies

**Figure 2:**
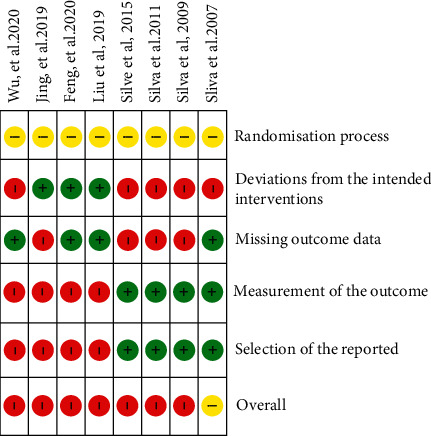
Risk of bias in included studies.

The results of bias testing are shown in Figure 2. Most studies had a high risk of bias. Although the majority of studies are reported using a randomization process, all studies were evaluated as “unclear” because they did not include information about the method of concealment. There was a high risk of deviation from treatment in parent-delivered massage interventions as strict control could not be enforced. Four studies were rated as “high bias” as over 10% of the participant data were missing in the experimental group and there were no reports of dropouts [21, 22, 24, 28]. In massage trials, it is difficult to blind participants and therapists. The assessors were blinded by professional examiners [27, 30] or blinded teachers [28, 29] and were rated as “low bias” in terms of outcome. No information about the outcome assessors was available in the Tui na studies, and they were, therefore, evaluated as “high bias” in this domain. Moreover, the Tui na studies also showed “high bias” in multiple analyses of the effective rate based on different evaluation standards of total score change [31–33] and in reports of only a subset [32].

### 3.4. Main Massage Intervention and Massage Techniques

Four studies used Qigong massage, the protocol consisted of 12 movements that followed the front and back of the body along acupuncture channels. The massage proceeds from the top of the head to the torso and the toes and includes tapping on the head and face, patting the torso on both sides, rubbing the abdomen, pressing the upper and lower limbs, twirling-rotating the fingers and toes, and stretching the lower limbs [35, 36]. Patting, pressing, finger-tapping, rubbing, and squeezing are the most used techniques in Qigong massage and can be safely administered by therapists and trained parents [27–30]. Tui na intervention was used by massage therapists in the remaining four studies, which incorporates pushing and kneading techniques, focusing on the head, hands, abdomen, and back [31–34], and involved selected acupoints Zanzhu (BL2), Geisoma, Yamen (GV15), Taiyang (EX-HN5), Wangu (GB12), Fengchi (GB20), Tianshu (ST25), Guanyuan (CV4), and Zusanli (ST36).

The duration and frequency of massage interventions varied widely, ranging from once a day to twice a week, for a duration of 15–30 mins, and lasted for six weeks to five months, and only one study had reported on the maintenance of efficacy after five months of massage treatment, suggesting that there was no significant mean difference after follow-up [28].

### 3.5. Main Evaluation Tools and Results of Intervention

#### 3.5.1. ABC and CARS

The majority of studies evaluated outcomes using ABC and CARS, both of which are observational rating scales to identify and characterize ASD. In these studies, they are often filled out by parents, teachers, or therapists who have difficulty in blinding. The ABC evaluates typical features of ASD across five domains: sensory, relating, body and object use, language, and social and self-help. It is intended to be used by a parent or a teacher who has known the child for at least three to six weeks [37]. CARS is broadly used by professionals to diagnose ASD. It consists of 15 domains for assessing behavior [38], including relation to people, imitation, emotional response, body use, fear or nervousness, activity level, and general impressions. The total raw score in the ABC and CARS was used to determine the severity of autism, where higher scores indicated more severe levels; however, studies have reported inconsistencies in their judgments [39, 40].

#### 3.5.2. Evaluation Results of the Massage Intervention

Seven studies used the ABC. Five of these studies reported significant total score reductions in the massage group versus the control group [21, 22, 24, 25, 27], which means there is a decrease in the severity of autism. The size of the effect was medium in some studies [21, 22, 24]: one study reported no significant difference [27] and one study used only the self-help score [32]. Most studies reported ABC total scores before and after intervention with subset scores being ignored. Liu et al. [31] reported a significant decrease in the sensory subset score but no significant changes were observed in the other domains. Two studies mentioned CARS scores; one reported no significant decline in the total score (*p*=0.071) after Qigong massage [30], while Feng et al. [33] found a significantly reduced total score (*p* < 0.05).

Although the results showed that massage therapy improved overall ABC and CARS scores, the data failed to identify improvement in specific subset symptoms or the degree to which symptoms were improved.

### 3.6. Safety

One study reported no adverse effects of massage therapy conducted on children with ASD [30]. The other studies reported no safety issues.

## 4. Discussion

This review identified eight RCTs examining the efficacy of massage interventions on children with ASD. Our review mainly summarized five aspects of these studies: the main kinds of massage treatment, the effect of massage on overall symptoms of ASD (evaluated with overall scores on ABC and CARS scales), the greatest improvement in specific symptom categories of ASD, the intervention and follow-up periods after massage therapy, and the overall safety of massage therapy.

Within those studies, we observed Qigong massage and Tui na were used as interventions. Qigong massage such as Tui na was also under the theoretical framework of traditional Chinese medicine. All reviewed articles reported significant improvements of symptoms in children with ASD who received massage, especially in the sensory domain, and all studies reported that massage in combination with the control group treatment was superior to control treatment alone. The intervention cycle ranged from once a day to twice a week, for a duration of 15–30 mins and lasted for six weeks to five months.

Moreover, from the studies that we reviewed, outcome measures only described improvement in the symptoms of ASD but did not explore possible neural mechanisms. From the perspective of modern medicine, massage is a touch-based therapy [41]. Touch can produce a variety of stimuli, such as pressure, pain, temperature changes, and muscle movement [42]; however, the effect of touch on brain activity remains unknown. According to functional magnetic resonance imaging data, massage not only modulates areas of the brain associated with arousal and consciousness (i.e., insula, anterior cingulate, posterior cingulate, inferior parietal cortices, and medial prefrontal cortices) [43] but also influences several brain regions associated with stress and emotion regulation, such as the amygdala, anterior cingulate cortex, and hypothalamus [36]. Morhenn et al. [44] found that massage therapy increased oxytocin while decreasing adrenocorticotropin, nitric oxide, and beta-endorphin, which may explain how social connections reduce morbidity and mortality. Changes in salivary oxytocin levels were observed in seven children with ASD (aged 8–12 years old) after mother-delivered massage therapy [39], certifying the relationship between touch and oxytocin concentration. Li et al. [45] indicated that improvement of oxytocin levels and the neural circuits involved in social interaction could be therapeutic for ASD. In a pilot study (not RCT design) by Jerger et al. [46], heart rate variability (HRV) and cerebral oximetry of the prefrontal cortex were used to explore the neural mechanisms of Qigong massage intervention in 20 children with ASD.

All reviewed articles reported significant improvements of symptoms in children with ASD who received massage. Moreover, massage treatment in combination with control group treatment was superior to control treatment alone. No adverse effects were reported in any of the studies examined. Overall, the evidence seems to indicate that massage therapy was an effective and safe option for treating symptoms of ASD. However, these conclusions must be interpreted cautiously due to the following conditions. *Quality of study*: the majority of studies were at high risk of bias, and low quality of evidence makes it difficult to conclude that massage is effective for ASD.

### 4.1. Massage Treatment

Interventions in the experiment group were not limited to massage treatment alone but also included special education and behavioral interventions. The results concerning the efficacy of massage as monotherapy for each symptom of ASD are yet to be proven.

### 4.2. Massage Protocol

Although the massage treatment was based on traditional Chinese medicine principles, there were differences in the operation process of massage therapy, including the choice of massage technique and acupoints, and the length, frequency, and duration of treatment.

### 4.3. Outcome Measure

Subjective questionnaires were used in most of these studies, such as ABC and CARS. However, these scales were designed to diagnose ASD and not to measure treatment effectiveness. Furthermore, multiple scale subsets covered the same index but only the scale total score was reported. In some cases, only subsets such as sensory index were included in ABC, PDDBI, and SSC. This highlights a lack of objective measurement and minimal exploration of the mechanisms to improve core symptoms.

### 4.4. Blinding

Massage (especially parent-delivered massage) intervention is hard to blind participants and parents. Some studies used parents to assess ABC, while others failed to report results evaluated by blinded researchers.

### 4.5. Participants

Most studies focused on young children aged 3 to 6 years. From the baseline of ABC score, children were identified as mildly/moderately ASD. This conclusion was limited by the characteristics of the participants.

Our review had several limitations. First, our search strategy was restricted to articles in English and Chinese. Second, only a few RCT_S_ were eligible for inclusion in the study, and all of the Qigong massage studies were only reported by a single research team. Furthermore, both Qigong massage and Tui na interventions were based on the theoretical framework of traditional Chinese medicine. Finally, due to the heterogeneity of the control group interventions and the low quality of available studies, a meta-analysis was not possible to conduct.

## 5. Conclusions

Our review intended to further our understanding of the outcomes of massage therapy in the treatment of children with ASD. The results of the present review suggest that Qigong massage and Tui na in combination with their respective control group treatments were the major existing interventions; although the results of these studies seemed to be positive, the methodological flaws and heterogeneity in the study design made the efficacy of massage as an intervention for this patient population inconclusive. Future research studies need to include large sample sizes, double-blind design, appropriate outcome measures, long observation, long follow-up periods, and consensus on standardized treatment and additional therapies, in order to provide a better quality of evidence. Meanwhile, other types of massage interventions, as well as massage therapy for older children with ASD, will also be worth investigating.

## Figures and Tables

**Table 1:** Characteristics of the included studies.

Study	Participants	Intervention	Comparator	Outcome measures	Major findings
Silva et al. (2007) [27]	*N* = 15 (*M* = 13, *F* = 2), 3∼6 years old	EG: (*N* = 8) Qigong massage + State of Oregon's special education program, duration 5 months of treatment twice a week for two five-week periods, with five weeks in between, twice a week for 5 weeks (by a specialist), every day for 5 months (by a parent) 15 min/d	CG: (*N* = 7) state of Oregon's special education Program	(1) Batelle: the cognitive domain screening test, (2) Sensory profile evaluation tool, (3) Vineland adaptive behavior scales, (4) ABC (5) Parent questionnaire	(1) Compared with CG, significant improvement in the sensory profile score, sensory processing score, sensory modulation score, social skills, and the basic living skills score in EG was observed, and autism behavior decreased in both groups (2) No statistical difference was observed between motor and language development. Improvement in bowel and sleep abnormalities in EG was observed

Silva et al. (2009) [28]	*N* = 46 (*M* = 37, *F* = 9), 3∼6 years old	RCT EG: (*N* = 25) Qigong massage plus special education program once a week (by a therapist) and everyday for 5 months (by a parent) 15 min/d	CG: (*N* = 21) special education program	(1) PDDBI teacher and parent versions, (2) ABC, and (3) SSC	(1) Compared with CG, significant main intervention effects were found in the ABC and the PDDBI social/communication composite. Significant main intervention effects were found in parent maladaptive behavior composite and PDDBI social/Communication composite (2) No significant treatment effect was found on the PDDBI measure of maladaptive classroom behavior between the two groups as both were being improved (3) Sense and self-regulation impairment (SSC and PDDBI sensory) and the three composite scores of the PDDBI (the maladaptive behavior composite, social/communication composite, and the total autism composite) were highly related
Silva et al. (2011) [29]	*N* = 47 (*M* = 33, *F* = 14), 3∼6 years old	RCT EG: (*N* = 28) trained parent Qigong massage plus special education program everyday for 5 months (by a parent) 15 min/d	CG: special education program (*N* = 19)	(1) ABC, (2) PDDBI, (3) APSI, and (4) SSC	Compared with CG, significant intervention effects were found for the PDDBI, the SSC, and the APSI. The main effect was found in the interaction between treatment and severity of sensory and self-regulation impairment on the PDDBI scales
Silva et al. (2015) [30]	*N* = 84 (*M* = 75, *F* = 9), 3∼6 years old	RCT EG: (*N* = 42) Qigong massage plus special education program once a week (by a therapist) and everyday (by a parent) 15 min/d RCT	CG: (*N* = 42) special education program	(1) CARS-2edition, (2) PLS-5edition, (3) Vineland-II, (4) ABC, (5) SSC, (6) APSI, and (7) The beach center family-professional partnership scale	Compared with CG, significant improvement in normalization of receptive language, autistic behavior, total sensory abnormalities, tactile abnormalities, and decreased autism severity was observed. Parents reported improved child-to-parent interactions, bonding, and significant decreased parenting stress

Liu et al. (2017) [31]	*N* = 60 (*M* = 50, *F* = 10) 2–8 years old	EG: (*N* = 30) acupuncture plus Tui na (by a specialist), acupuncture for 1 hour, 2 times a week for 30 times; Tui na, 2 times a week for30 times	CG:(*N* = 30) acupuncture (by a specialist)	(1) ABC	(1) Compared with CG, significant improvement in the ABC total scores in both groups before and after treatment was observed, and the EG group showed an increased difference in the ABC total scores (2) EG: significant improvement in four of the ABC scale dimensions (sensation, communication, physical movement, and self-care ability) except language was observed (3) CG: the ABC scale showed significant improvement in other dimensions except for sensation

Jing et al. (2019) [32]	*N* = 136 *M* = 109, *F* = 27 age:4.42 ± 0.19	RCT EG:(*N* = 68)Tui na plus acupuncture (by a specialist) 2 section/week, 15 weeks	CG: (*N* = 68) acupuncture (by a specialist) 2 section/week, 15 weeks	(1) ABC subset self-help	(1) After the intervention, the self-help score in both groups decreased, and EG was significantly better than that of CG (2) The effective rate of the EG (100%) was higher than that of CG (96.43%) but showed no significant difference

Feng et al. (2020) [33]	*N* = 44 (*M* = 33, *F* = 11), age:3.57 ± 0.84	RCT EG: (*N* = 22) behavioral intervention plus traditional Chinese massage (by a specialist) 1 section/day, 5 section/week, 6 weeks.	CG: (*N* = 22) behavioral intervention (by a specialist)	(1) ABC (2) CARS	After the intervention, the ABC and CARS score in both groups decreased, and EG was significantly better than CG

Wu et al. 2020 [34]	*N* = 120 (*M* = 57, *F* = 63) Age: 2.69 ± 0.22	RCT EG: (*N* = 60)Tui na plus acupuncture (by a specialist) 2 section/day, 30 mins	CG: (*N* = 60) Acupuncture (by a specialist)	(1) Language ability (self-designed questionnaire) (2) SAS, (3) SDS 4. Parents' treatment satisfaction	(1) EG language recovery level and language training effect were significantly higher than that of the control group (2) The scores of anxiety and depression in EG were lower than those of the CG (*p* < 0.01) (3) The treatment satisfaction of EG was higher than that of CG (0 < 0.05)

## Data Availability

This is a systematic review. No new data were generated.

## Supplementary Materials

Supplementary Materials PRISMA 2020 checklist.
